# Targeting HIF2–PTHrP axis in kidney cancer: a paradigm shift in therapy?

**DOI:** 10.1093/ckj/sfag074

**Published:** 2026-03-05

**Authors:** Eva Pella, Maria Jose Soler

**Affiliations:** Nephrocare Hemodialysis Unit, Thessaloniki, Greece; Nephrology department, Vall d’Hebron Hospital University. Nephrology and transplantation group. Vall d’Hebron Institut de Recerca, Vall d’Hebron Barcelona Hospital Campus, Barcelona, Spain; Redes de Investigación Cooperativa Orientada a Resultados en Salud, RICORS2040 (RD24/0004/0031)

Cancer cachexia and hypercalcemia are well-recognized components of the paraneoplastic syndrome in kidney cancer, including the clear cell renal cell carcinoma (ccRCC). These complications substantially diminish quality of life, impair tolerance to anticancer therapies, and contribute to poor survival [1–3]. Despite their clinical impact, the molecular mechanisms underlying most of these systemic complications remain poorly understood, and current management strategies are tumor-centric rather than symptom-oriented [1, 3].

The suggested initiating molecular event in ccRCC is the loss of the von Hippel–Lindau (pVHL) protein function, which leads to the stabilization of the Hypoxia-Inducible Factor-2 alpha (HIF2α) and the transcriptional upregulation of the PTHLH gene [4] (Figure 1). In a recent study published in *Nature Medicine* [1], the authors not only strengthened the earlier conclusion that PTHLH is a direct HIF2 target gene [5], but they also provided further evidence that its product, the parathyroid hormone-related protein (PTHrP) should also be considered a potential treatment target for managing paraneoplastic metabolic complications.

**Figure 1:**
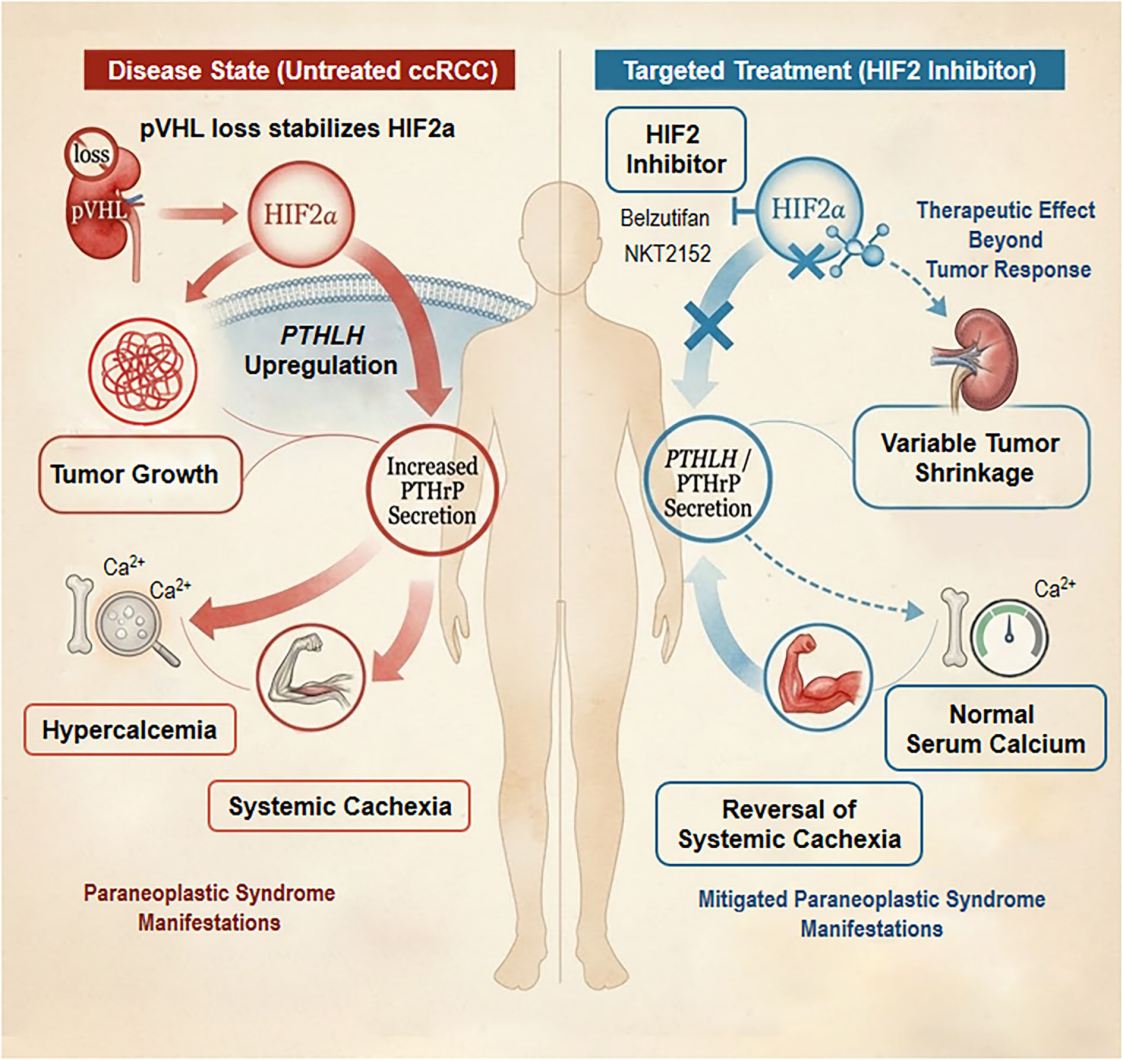
The HIF2–PTHrP axis in untreated clear cell renal cell carcinoma (ccRCC) and after targeted HIF2 Inhibitor treatment.

They used orthotopic xenograft models and patients with ccRCC, showing that elevated PTHrP was both necessary and sufficient to trigger systemic cachexia and hypercalcemia, independently of tumor size. Notably, HIF2 inhibition with pharmacologic agents reversed established cachexia, preserved lean and fat mass, and rapidly normalized serum calcium levels, even in cases without tumor shrinkage. Clinically, treatment with HIF2 inhibitors such as Belzutifan and NKT2152 led to rapid reductions in circulating PTHrP, weight gain and improved metabolic parameters, even among patients without radiographic tumor response [1] (Figure 1).

## CLINICAL IMPLICATIONS

This study provides a mechanistic redefinition of cancer-associated cachexia and hypercalcemia in ccRCC, shifting the paradigm from a traditionally perceived nonspecific systemic metabolic syndrome driven by inflammatory cytokines, energy imbalance, and tumor burden to a targetable endocrine process [1, 3, 6]. By indicating the HIF2–PTHrP axis as a driver of these metabolic complications, this study challenges the conventional approach of relying on tumor shrinkage as the primary therapeutic endpoint and supports the inclusion of paraneoplastic complications amelioration as outcomes in ccRCC trials. In clinical practice, nephrologists and oncologists may now monitor PTHrP levels and implement HIF2 inhibition to alleviate cancer-related wasting and hypercalcemia, ultimately aiming to improve patient quality of life independently of radiographic tumor shrinkage. Moreover, these findings introduce a precision-based approach to the management of paraneoplastic syndromes in ccRCC by suggesting that HIF2 inhibition may serve a dual role as both anticancer and symptom-directed therapy [1]. Nonetheless, important questions remain regarding the durability of cachexia reversal, applicability across disease stages and the potential interaction with chronic kidney disease, anemia and mineral metabolism disorders. Overall, targeting the HIF2–PTHrP axis represents a paradigm shift toward mechanism-based, symptom-oriented management of paraneoplastic complications in kidney cancer, reinforcing a strategy aimed at improving paraneoplastic complications and quality of life, not solely tumor control.
